# Nonlinear Hebbian Learning as a Unifying Principle in Receptive Field Formation

**DOI:** 10.1371/journal.pcbi.1005070

**Published:** 2016-09-30

**Authors:** Carlos S. N. Brito, Wulfram Gerstner

**Affiliations:** 1 School of Computer and Communication Sciences and School of Life Science, Brain Mind Institute, Ecole Polytechnique Federale de Lausanne, Lausanne EPFL, Switzerland; 2 Gatsby Computational Neuroscience Unit, University College London, London, United Kingdom; Duke University, UNITED STATES

## Abstract

The development of sensory receptive fields has been modeled in the past by a variety of models including normative models such as sparse coding or independent component analysis and bottom-up models such as spike-timing dependent plasticity or the Bienenstock-Cooper-Munro model of synaptic plasticity. Here we show that the above variety of approaches can all be unified into a single common principle, namely nonlinear Hebbian learning. When nonlinear Hebbian learning is applied to natural images, receptive field shapes were strongly constrained by the input statistics and preprocessing, but exhibited only modest variation across different choices of nonlinearities in neuron models or synaptic plasticity rules. Neither overcompleteness nor sparse network activity are necessary for the development of localized receptive fields. The analysis of alternative sensory modalities such as auditory models or V2 development lead to the same conclusions. In all examples, receptive fields can be predicted a priori by reformulating an abstract model as nonlinear Hebbian learning. Thus nonlinear Hebbian learning and natural statistics can account for many aspects of receptive field formation across models and sensory modalities.

## Author Summary

The question of how the brain self-organizes to develop precisely tuned neurons has puzzled neuroscientists at least since the discoveries of Hubel and Wiesel. In the past decades, a variety of theories and models have been proposed to describe receptive field formation, notably V1 simple cells, from natural inputs. We cut through the jungle of candidate explanations by demonstrating that in fact a single principle is sufficient to explain receptive field development. Our results follow from two major insights. First, we show that many representative models of sensory development are in fact implementing variations of a common principle: nonlinear Hebbian learning. Second, we reveal that nonlinear Hebbian learning is sufficient for receptive field formation through sensory inputs. The surprising result is that our findings are robust of specific details of a model, and allows for robust predictions on the learned receptive fields. Nonlinear Hebbian learning is therefore general in two senses: it applies to many models developed by theoreticians, and to many sensory modalities studied by experimental neuroscientists.

## Introduction

Neurons in sensory areas of the cortex are optimally driven by stimuli with characteristic features that define the receptive field of the cell. While receptive fields of simple cells in primary visual cortex (V1) are localized in visual space and sensitive to the orientation of light contrast [1], those of auditory neurons are sensitive to specific time-frequency patterns in sounds [2]. The concept of a receptive field is also useful when studying higher-order sensory areas, for instance when analyzing the degree of selectivity and invariance of neurons to stimulus properties [3, 4].

The characteristic receptive fields of simple cells in V1 have been related to statistical properties of natural images [5]. These findings inspired various models, based on principles as diverse as sparse sensory representations [6], optimal information transmission [7], or synaptic plasticity [8]. Several studies highlighted possible connections between biological and normative justifications of sensory receptive fields [9, 10, 11, 12], not only in V1, but also in other sensory areas [13], such as auditory [14, 15] and secondary visual cortex (V2) [16].

Since disparate models appear to achieve similar results, the question arises whether there exists a general underlying concept in unsupervised learning models [15, 17]. Here we show that the principle of nonlinear Hebbian learning is sufficient for receptive field development under rather general conditions. The nonlinearity is defined by the neuron’s f-I curve combined with the nonlinearity of the plasticity function. The outcome of such nonlinear learning is equivalent to projection pursuit [18, 19, 20], which focuses on features with non-trivial statistical structure, and therefore links receptive field development to optimality principles.

Here we unify and broaden the above concepts and show that plastic neural networks, sparse coding models and independent component analysis can all be reformulated as nonlinear Hebbian learning. For natural images as sensory input, we find that a broad class of nonlinear Hebbian rules lead to orientation selective receptive fields, and explain how seemingly disparate approaches may lead to similar receptive fields. The theory predicts the diversity of receptive field shapes obtained in simulations for several different families of nonlinearities. The robustness to model assumptions also applies to alternative sensory modalities, implying that the statistical properties of the input strongly constrain the type of receptive fields that can be learned. Since the conclusions are robust to specific properties of neurons and plasticity mechanisms, our results support the idea that synaptic plasticity can be interpreted as nonlinear Hebbian learning, implementing a statistical optimization of the neuron’s receptive field properties.

## Results

### The effective Hebbian nonlinearity

**Fig 1:**
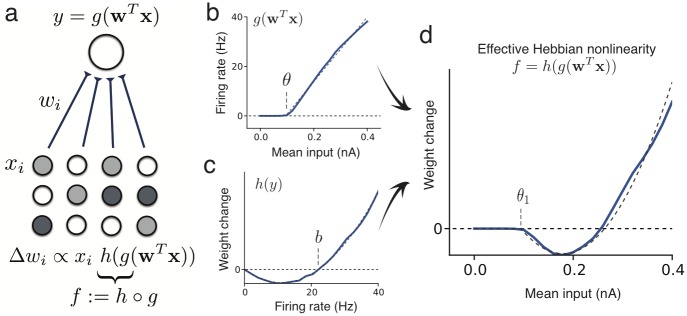
The effective Hebbian nonlinearity of plastic cortical networks. (**a**) Receptive field development between an input layer of neurons with activities *x*_*i*_, connected by synaptic projections *w*_*i*_ to a neuron with firing rate *y*, given by an f-I curve *y* = *g*(**w**^*T*^**x**)). Synaptic connections change according to a Hebbian rule Δ*w*_*i*_ ∝ *x*_*i*_ *h*(*y*). (**b**) f-I curve (blue) of a GIF model [26] of a pyramidal neurons in response to step currents of 500 ms duration (dashed line: piece-wise linear fit, with slope *a* = 143 Hz/nA and threshold *θ* = 0.08 nA). (**c**) Plasticity function of the triplet STDP model [24] (blue), fitted to visual cortex plasticity data [27, 24], showing the weight change Δ*w*_*i*_ as a function of the post-synaptic rate *y*, under a constant pre-synaptic stimulation *x*_*i*_ (dashed line: fit by quadratic function, with LTD factor *b* = 22.1 Hz). (**d**) The combination of the f-I curve and plasticity function generates the effective Hebbian nonlinearity (dashed line: quadratic nonlinearity with LTD threshold *θ*_1_ = 0.08 nA, LTP threshold *θ*_2_ = 0.23 nA).

In classic rate models of sensory development [21, 8, 6], a first layer of neurons, representing the sensory input **x**, is connected to a downstream neuron with activity *y*, through synaptic connections with weights **w** (Fig 1a). The response to a specific input is *y* = *g*(**w**^*T*^ **x**), where *g* is the frequency-current (f-I) curve. In most models of Hebbian plasticity [22, 23], synaptic changes Δ**w** of the connection weights depend on pre- and post-synaptic activity, with a linear dependence on the pre-synaptic and a nonlinear dependence on the post-synaptic activity, Δ**w** ∝ **x** *h*(*y*), in accordance with models of pairing experiments [24, 10]. The learning dynamics arise from a combination of the neuronal f-I curve *y* = *g*(**w**^**T**^**x**) and the Hebbian plasticity function Δ**w** ∝ **x** *h*(*y*):

$$\Delta w\propto x\;h\left(g\left(w^{T}x\right)\right)\;=\;x\;f\left(w^{T}x\right)$$ (1)

where we define the *effective Hebbian nonlinearity* *f* ≔ *h* ∘ *g* as the composition of the nonlinearity in the plasticity rule and the neuron’s f-I curve. In an experimental setting, the pre-synaptic activity *x* is determined by the set of sensory stimuli (influenced by, e.g., the rearing conditions during sensory development [25]). Therefore, the evolution of synaptic strength, Eq 1, is determined by the effective nonlinearity *f* and the statistics of the input **x**.

Many existing models can be formulated in the framework of Eq 1. For instance, in a classic study of simple-cell formation [8], the Bienenstock-Cooper-Munro (BCM) model [22] has a quadratic plasticity nonlinearity, *h*_*θ*_(*y*) = *y*(*y* − *θ*), with a variable plasticity threshold *θ* = 〈*y*^2^〉, and a sigmoidal f-I curve, *y* = *σ*(**w**^*T*^ **x**). Since the threshold *θ* is adapted on a time scale sufficiently slow to sample the statistics of 〈*y*^2^〉 [28], and on a time scale faster than the learning dynamics [29], we may consider it as fixed, and the dynamics are well described by nonlinear Hebbian learning, Δ**w** ∝ **x** *h*_*θ*_(*σ*(**w**^*T*^**x**)), with a nonlinearity modulated by *θ*.

More realistic cortical networks have dynamical properties which are not accounted for by rate models. By analyzing state-of-the-art models of cortical neurons and synaptic plasticity, we inspected whether plastic spiking networks can be reduced to nonlinear Hebbian learning. We considered a generalized leaky integrate-and-fire model (GIF), which includes adaptation, stochastic firing and predicts experimental spikes with high accuracy [26], and we approximate its f-I curve by a linear rectifier, *g*(*u*) = *a*(*u* − *θ*)_+_, with slope *a* and threshold *θ* (Fig 1b).

As a phenomenological model of synaptic plasticity grounded on experimental data [27], we implemented triplet spike-timing dependent plasticity (STDP) [24]. In this STDP model, the dependence of long-term potentiation (LTP) upon two post-synaptic spikes induces in the corresponding rate model a quadratic dependence on the post-synaptic rate, while long-term depression (LTD) is linear. The resulting rate plasticity [24] is *h*(*y*) = *y*^2^ − *by*, with an LTD factor *b* (post-synaptic activity threshold separating LTD from LTP, Fig 1c), similar to the classic BCM model [22, 8].

Composing the f-I curve of the GIF with the *h*(*y*) for the triplet plasticity model, we have an approximation of the effective learning nonlinearity *f* = *h* ∘ *g* in cortical spiking neurons (Fig 1d), that can be described as a quadratic rectifier, with LTD threshold given by *θ*_1_ = *θ* and LTP threshold given by *θ*_2_ = *θ*+*b*/*a*. Interestingly, the f-I slope *a* and LTD factor *b* are redundant parameters of the learning dynamics: only their ratio counts in nonlinear Hebbian plasticity. Metaplasticity can control the LTD factor [24, 30], thus regulating the LTP threshold.

**Fig 2:**
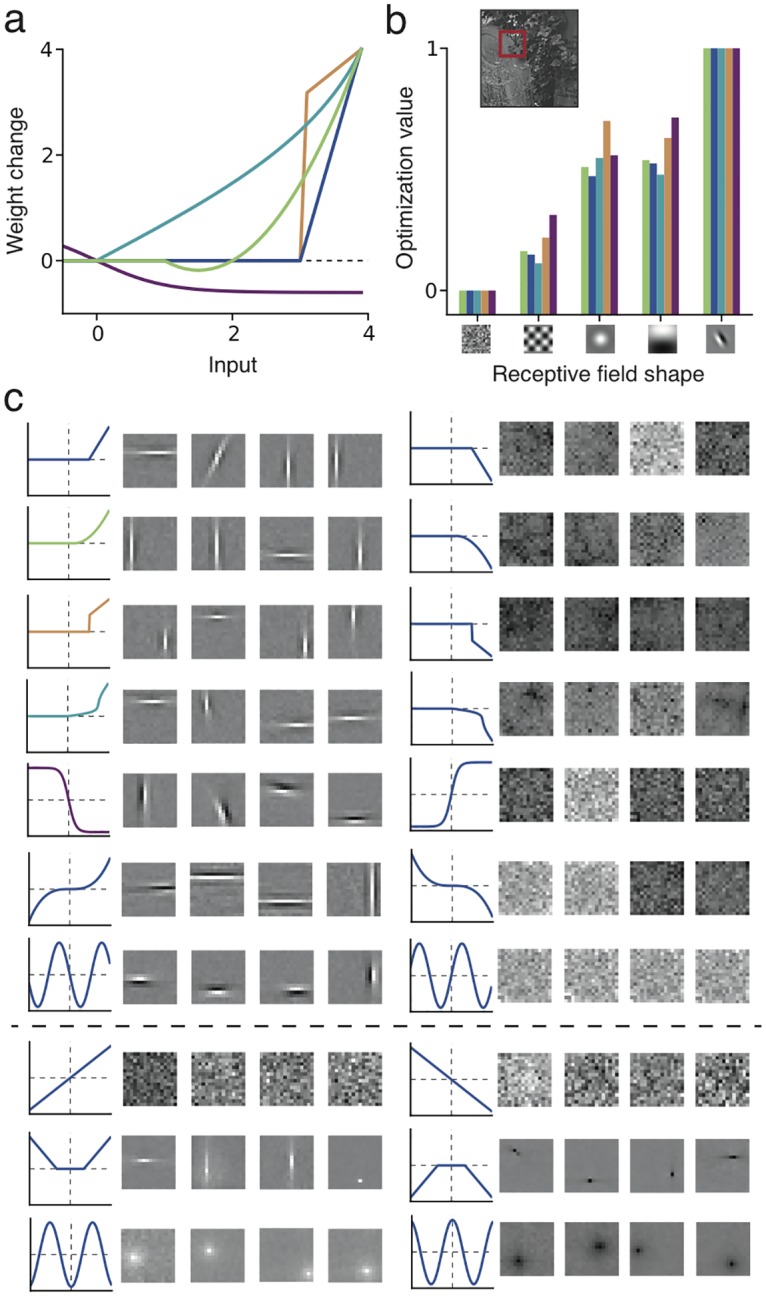
Simple cell development from natural images regardless of specific effective Hebbian nonlinearity. (**a**) Effective nonlinearity of five common models (arbitrary units): quadratic rectifier (green, as in cortical and BCM models, *θ*_1_ = 1., *θ*_2_ = 2.), linear rectifier (dark blue, as in *L*_1_ sparse coding or networks with linear STDP, *θ* = 3.), Cauchy sparse coding nonlinearity (light blue, *λ* = 3.), *L*_0_ sparse coding nonlinearity (orange, *λ* = 3.), and negative sigmoid (purple, as in ICA models). (**b**) Relative optimization value 〈*F*(**w**^*T*^**x**)〉 for each of the five models in **a**, for different preselected features **w**, averaged over natural image patches ****x****. Candidate features are represented as two-dimensional receptive fields. For all models, the optimum is achieved at the localized oriented receptive field. Inset: Example of natural image and image patch (red square) used as sensory input. (**c**) Receptive fields learned in four trials for ten effective Hebbian functions *f* (from top: the five functions considered above, *u*^3^, − *sin*(*u*), *u*, (|*u*| − 2)_+_, − *cos*(*u*)) (**left** **column**), and their opposites − *f* (**right column**). The first seven functions (above the dashed line) lead to localized oriented filters, while a sign-flip leads to random patterns. Linear or symmetric functions are exceptions and do not develop oriented filters (**bottom** **rows**).

If one considers a linear STDP model [31, 32] instead of the triplet STDP [24], the plasticity curve is linear [23], as in standard Hebbian learning, and the effective nonlinearity is shaped by the properties of the f-I curve (Fig 2a).

In the following we consider these rate approximations of STDP and analyze the developmental properties of spiking neurons through their effective nonlinearities.

### Sparse coding as nonlinear Hebbian learning

Beyond phenomenological modeling, normative principles that explain receptive fields development have been one of the goals of theoretical neuroscience [33]. Sparse coding [6] starts from the assumptions that V1 aims at maximizing the sparseness of the activity in the sensory representation, and became a well-known normative model to develop orientation selective receptive fields [9, 12, 13]. We demonstrate that the algorithm implemented in the sparse coding model is in fact a particular example of nonlinear Hebbian learning.

The sparse coding model aims at minimizing an input reconstruction error $E\;=\;\frac{1}{2}||x\;-\;{Wy||}^{2}\;+\;\lambda S\left(y\right)$, under a sparsity constraint *S* with relative importance *λ* > 0. For *K* hidden neurons *y*_*j*_, such a model implicitly assumes that the vector **w**_**j**_ of feed-forward weights onto neuron *j* are mirrored by hypothetical “reconstruction weights”, **W** = [**w**_1_ … **w**_*K*_]. The resulting encoding algorithm can be recast as a neural model [34], if neurons are embedded in a feedforward model with lateral inhibition, **y** = *g*(**w**^*T*^**x** − **v**^*T*^**y**), where *v* are inhibitory recurrent synaptic connections (see Methods). In the case of a single output neuron, its firing rate is simply *y* = *g*(**w**^*T*^**x**). The nonlinearity *g* of the f-I curve is threshold-like, and determined by the choice of the sparsity constraint [34], such as the Cauchy, *L*_0_, or *L*_1_ constraints (Fig 2a, see Methods).

If weights are updated through gradient descent so as to minimize *E*, the resulting plasticity rule is Oja’s learning rule [35], Δ**w** ∝ **x** *y* − **w** *y*^2^. The second term −**w** *y*^2^ has a multiplicative effect on the strength of synapses projecting onto the same neuron (weight rescaling), but does not affect the receptive field shape, whereas the first term **x** *y* drives feature selectivity and receptive field formation.

Together, these derivations imply that the one-unit sparse coding algorithm can be implemented by an effective nonlinear Hebbian rule combined with weight normalization. Although the plasticity mechanism is linear, Δ**w** ∝ **x** *y*, a nonlinearity arises from the f-I curve, *y* = *g*(**w**^*T*^**x**), so that the effective plasticity is

$$\Delta w\propto x\;g(w^{T}x)$$ (2)

This analysis reveals an equivalence between sparse coding models and neural networks with linear plasticity mechanisms, where the sparsity constraint is determined by the f-I curve *g*.

While Oja’s rule is commonly associated with principal component analysis (PCA), developing connections that project the input in the direction of largest variance [35], this relation is only valid for linear neurons. When nonlinear neurons are considered, Oja’s rule is also sensitive to higher-order statistics, as analyzed below.

Similarly, algorithms performing independent component analysis (ICA), a model class closely related to sparse coding, also perform effective nonlinear Hebbian learning, albeit inversely, with linear neurons and a nonlinear plasticity rule [36]. For variants of ICA based on information maximization [7] or kurtosis [36] different nonlinearities arise (Fig 2a), but Eq 2 applies equally well. Hence, various instantiations of sparse coding and ICA models not only relate to each other in their normative assumptions [37], but when implemented as iterative gradient update rules, they all employ nonlinear Hebbian learning.

### Simple cell development for a large class of nonlinearities

Since the models described above can be implemented by similar plasticity rules, we hypothesized nonlinear Hebbian learning to be a general principle that explains the development of receptive field selectivity. Nonlinear Hebbian learning with an effective nonlinearity *f* is linked to an optimization principle with a function $F\left(u\right)\;=\;\int_{0}^{u}f\left(u^{\prime }\right)du^{\prime }$ [19, 20]. For an input ensemble **x**, optimality is achieved by weights $\tilde{w}$ that maximize $\langle F\left(\tilde{w}{}^{T}x\right)\rangle$, where angular brackets denote the average over the input statistics. Nonlinear Hebbian learning is a stochastic gradient ascent implementation of this optimization process, called projection pursuit [18, 19, 20]:

$$\tilde{w}=max_{w}\langle F\left(w^{T}x\right)\rangle \Rightarrow \;\Delta \;w\propto x\;f\left(w^{T}x\right)$$ (3)

Motivated by results from ICA theory [38] and statistical properties of whitened natural images [5], we selected diverse Hebbian nonlinearities *f* (Fig 2a) and calculated the corresponding optimization value 〈*F*(**w**^*T*^**x**)〉 for different features of interest that we consider as candidate RF shapes, with a whitened ensemble of patches extracted from natural images as input (see Methods). These include a random connectivity pattern, a non-local oriented edge (as in principal components of natural images) and localized oriented edges (as in cat and monkey simple cells in the visual cortex), shown in Fig 2b. The relative value of 〈*F*(**w**^*T*^**x**)〉 between one feature and another was remarkably consistent across various choices of the nonlinearity *f*, with localized orientation-selective receptive fields as maxima (Fig 2b). Furthermore, we also searched for the maxima through gradient ascent, so as to confirm that the maxima are orientation selective (Fig 2c, **left**). Our results indicate that receptive field development of simple cells is mainly governed by the statistical properties of natural images, while robust to specific model assumptions.

**Fig 3:**
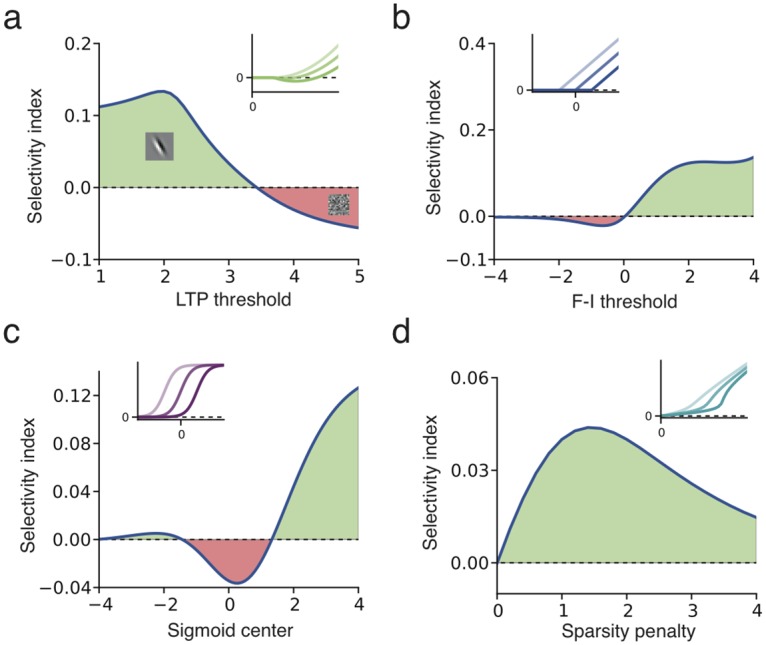
Selectivity index for different nonlinearities *f*. (**a**) Quadratic rectifier (small graphic, three examples with different LTP thresholds) with LTD threshold at *θ*_1_ = 1: LTP threshold must be below 3.5 to secure positive selectivity index (green region, main Fig) and learn localized oriented receptive fields (inset). A negative selectivity index (red region) leads to a random connectivity pattern (inset) (**b**) Linear rectifier: activation threshold must be above zero. (**c**) Sigmoid: center must be below *a* = − 1.2 or, for a stronger effect, above *a* = +1.2. The opposite conditions apply to the negative sigmoid. (**d**) Cauchy sparse coding nonlinearity: positive but weak feature selectivity for any sparseness penalty *λ* > 0. Insets show the nonlinearities for different choices of parameters.

The relevant property of natural image statistics is that the distribution of a feature derived from typical localized oriented patterns has high kurtosis [5, 6, 39]. Thus to establish a quantitative measure whether a nonlinearity is suitable for feature learning, we define a *selectivity index* (*SI*), which measures the relative value of 〈*F*(.)〉 between a variable *l* with a Laplacian distribution and a variable *g* with Gaussian distribution [38]: *SI* = (〈*F*(*l*)〉 − 〈*F*(*g*)〉)/*σ*_*F*_ (see Methods). The Laplacian variable has higher kurtosis than the Gaussian variable, serving as a prototype of a kurtotic distribution. Since values obtained by filtering natural images with localized oriented patterns have a distribution with longer tails than other patterns [5], as does the Laplacian variable compared to the Gaussian, positive values *SI* > 0 indicate good candidate functions for learning simple cell-like receptive fields from natural images. We find that each model has an appropriate parameter range where *SI* > 0 (Fig 3). For example the quadratic rectifier nonlinearity needs an LTP threshold *θ*_2_ below some critical level, so as to be useful for feature learning (Fig 3a).

A sigmoidal function with threshold at zero has *negative SI*, but a *negative* sigmoid, as used in ICA studies [7], has *SI* > 0. More generally, whenever an effective nonlinearity *f* is not suited for feature learning, its opposite − *f* should be, since its *SI* will have the opposite sign (Fig 2c). This implies that, in general, half of the function space could be suitable for feature learning [38], i.e. it finds weights *w* such that the distribution of the feature **w**^*T*^**x** has a long tail, indicating high kurtosis (“kurtotic feature”). The other half of the function space learns the least kurtotic features (e.g. random connectivity patterns for natural images, Fig 2b and 2c).

This universality strongly constrains the possible shape of receptive fields that may arise during development for a given input dataset. For whitened natural images, a learnable receptive field is in general either a localized edge detector or a non-localized random connectivity pattern.

While there is no simple description for the class of suitable functions, we may gain some intuition by considering the class of rectified power functions, $F\left(u\right)=u_{+}^{r}$, *r* ∈ ℜ^+^. In the case of powers *r* > 2, the selectivity index is positive. As a consequence, any supra-linear nonlinearity $f\left(u\right)=u{}_{+}^{p}$ with *p* > 1 should be suitable for feature learning. In Table 1, we include the appropriate parameter range for various effective nonlinearities.

**Table 1:** Parameter ranges for suitable effective nonlinearities and corresponding optimization functions.

	Effective nonlinearity *f*(*u*)	Optimization function *F*(*u*)	Parameter range
Linear rectifier	(*u* − *θ*)_+_	${\left(u-\theta \right)}_{+}^{2}$	*θ* > 0
Quadratic rectifier	$-b\;{\left(u\;-\;1\right)}_{+}\;+\;{\left(u\;-\;1\right)}_{+}^{2}$	$-\frac{b}{2}{\left(u\;-\;1\right)}_{+}^{2}\;+\;\frac{1}{3}{\left(u\;-\;1\right)}_{+}^{3}$	*b* < 3.5
Sigmoid	(1 + *e*^ − 2(*u* − *a*)^)^ − 1^	$\frac{1}{2}\;log\left(1\;+\;e^{2(u-a)}\right)$	|*a*| > − 1.2
Negative sigmoid	−(1 + *e*^−2(*u*−*a*)^)^−1^	$-\frac{1}{2}\;log\left(1\;+\;e^{2(u-a)}\right)$	|*a*| < −1.2
Power	$u{}_{+}^{p}$	$\frac{1}{p+1}u{}_{+}^{p+1}$	*p* > 1

An important special case is an effective linear curve, *f*(*u*) = *u*, which arises when both f-I and plasticity curves are linear [21]. Because the linear model maximizes variance 〈(**w**^*T*^**x**)^2^〉, it can perform principal component analysis [35], but does not have any feature selectivity on whitened input datasets, where variance is constant (Fig 2c).

Symmetric effective nonlinearities, *f*(*u*) = *f*(−*u*), are also exceptions, since their corresponding optimization functions are asymmetric, *F*(*u*) = −*F*(−*u*), so that for datasets with symmetric statistical distributions, *P*(**x**) = *P*(−**x**), the optimization value will be zero, 〈*F*_*asym*._(**w**^*T*^**x**_*sym*._)〉 = 0. As natural images are not completely symmetric, localized receptive fields do develop, though without orientation selectivity, as illustrated by a cosine function and a symmetric piece-wise linear function as effective nonlinearities (Fig 2c, bottom rows).

### Predicting receptive field diversity

Sensory neurons display a variety of receptive field shapes [40], and modeling efforts [41, 9, 12] have attempted to understand the properties that give rise to the specific receptive fields seen in experiments. We show here that the shape diversity of a model can be predicted by our projection pursuit analysis, and is primarily determined by the statistics of input representation, while relatively robust to the specific effective nonlinearity.

**Fig 4:**
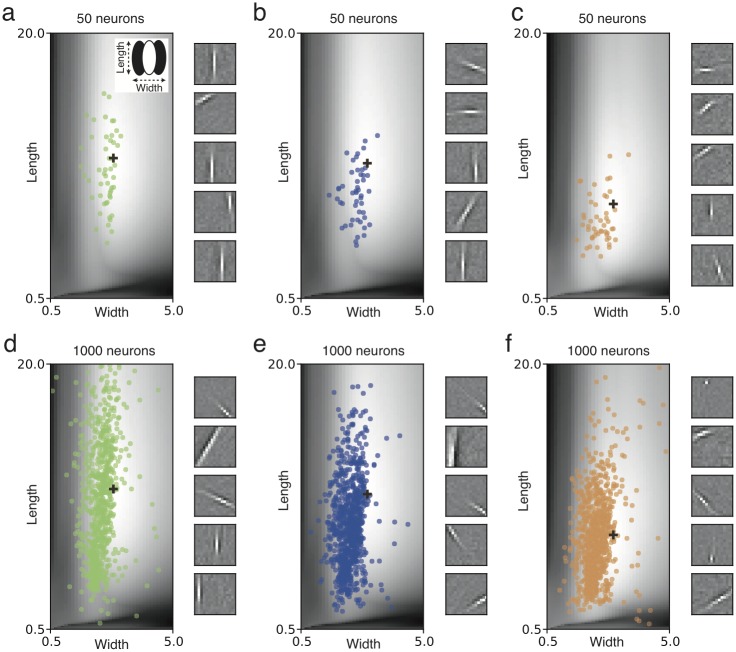
Optimal receptive field shapes in model networks induce diversity. (**a-f**) Gray level indicates the optimization value for different lengths and widths (see inset in **a**) of oriented receptive fields for natural images, for the quadratic rectifier (left, see Fig 2a), linear rectifier (center) and *L*_0_ sparse coding (right). Optima marked with a black cross. (**a-c**) Colored circles indicate the receptive fields of different shapes developed in a network of 50 neurons with lateral inhibitory connections. Insets on the right show example receptive fields developed during simulation. (**d-f**) Same for a network of 1000 neurons.

We studied a model with multiple neurons in the second layer, which compete with each other for the representation of specific features of the input. Each neuron had a piece-wise linear f-I curve and a quadratic rectifier plasticity function (see Methods) and projected inhibitory connections *v* onto all others. These inhibitory connections are learned by anti-Hebbian plasticity and enforce decorrelation of neurons, so that receptive fields represent different positions, orientations and shapes [42, 43, 44]. For 50 neurons, the resulting receptive fields became diversified (Fig 4a–4c, colored dots). In an overcomplete network of 1000 neurons, the diversity further increased (Fig 4d–4f, colored dots).

For the analysis of the simulation results, we refined our inspection of optimal oriented receptive fields for natural images by numerical evaluation of the optimality criterion 〈*F*(**w**^*T*^**x**)〉 for receptive fields **w** = **w**_*Gabor*_, described as Gabor functions of variable length, width and spatial frequency. For all tested nonlinearities, the optimization function for single-neuron receptive fields varies smoothly with these parameters (Fig 4, grey-shaded background). The single-neuron optimality landscape was then used to analyze the multi-neuron simulation results. We found that receptive fields are located in the area where the single-neuron optimality criterion is near its maximum, but spread out so as to represent different features of the input (Fig 4). Thus the map of optimization values, calculated from the theory of effective nonlinearity, enables us to qualitatively predict the shape diversity of receptive fields.

Although qualitatively similar, there are differences in the receptive fields developed for each model, such as smaller lengths for the *L*_0_ sparse coding model (Fig 4c). While potentially significant, these differences across models may be overwhelmed by differences due to other model properties, such as different network sizes or input representations.This is illustrated by observing that receptive field diversity for a given model differ substantially across network sizes (Fig 4).

**Fig 5:**
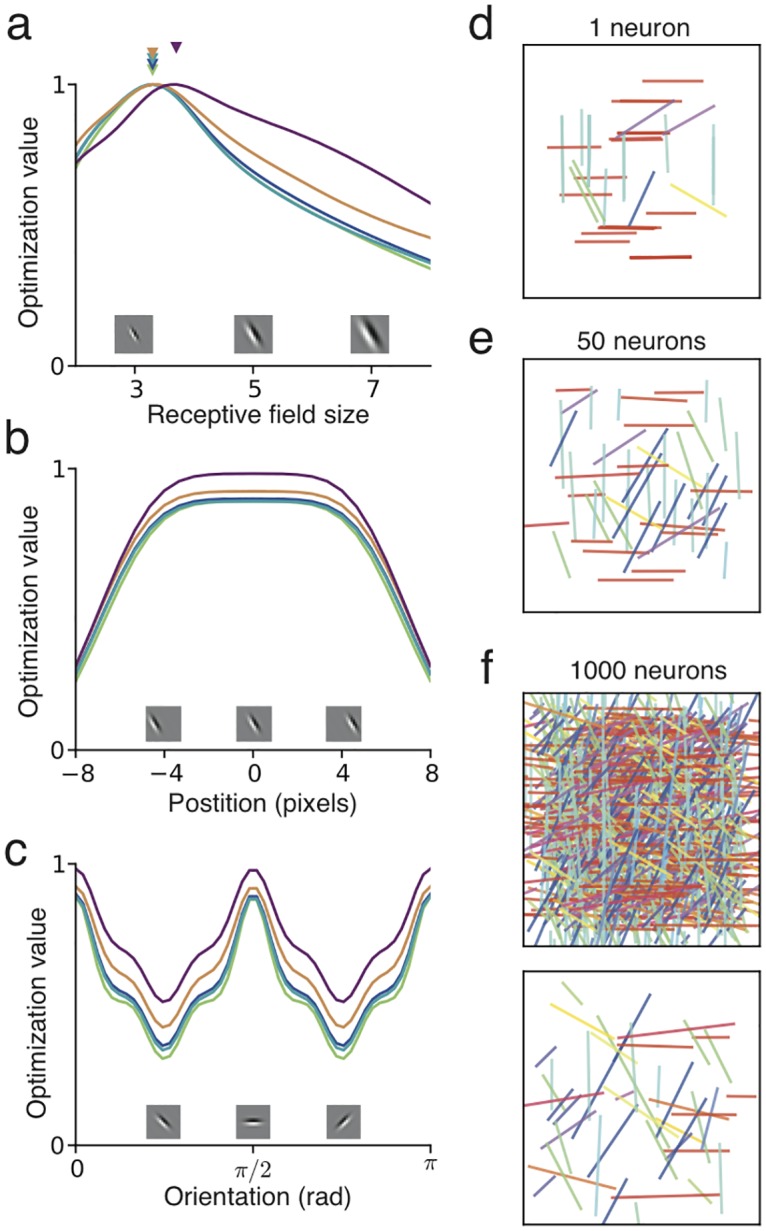
Diversity of receptive field size, position and orientation. (**a**) The optimization value of localized oriented receptive fields, within a 16x16 pixel patch of sensors, as a function of size (see Methods), for five nonlinearities (colors as in Fig 2a). Optimal size is a receptive field of width around 3 to 4 pixels (filled triangles). (**b**) The optimization value as a function of position of the receptive field center, for a receptive field width of 4 pixels, indicates invariance to position within the 16x16 patch, except near the borders. (**c**) The optimization value as a function of orientation shows preference toward horizontal and vertical directions, for all five nonlinearities. (**d**) Receptive field position, orientation and length (colored bars) learned for 50 single-neuron trials. The color code indicates different orientations. (**e**) Receptive field positions and orientations learned in a 50 neuron network reveal diversification of positions, except at the borders. (**f**) With 1000 neurons, positions and orientations cover the full range of combinations (top). Selecting 50 randomly chosen receptive fields highlights the diversification of position, orientation and size (bottom). Receptive fields were learned through the quadratic rectifier nonlinearity (*θ*_1_ = 1., *θ*_2_ = 2.).

We also studied the variation of receptive field position and orientation. For all five nonlinearities considered, the optimization value is equal for different positions of the receptive field centers, confirming the translation invariance in the image statistics, as long as the receptive field is not too close to the border of the anatomically allowed fan-in of synaptic connections (Fig 5b). Also, all nonlinearities reveal the same bias towards the horizontal and vertical orientations (Fig 5c). These optimality predictions are confirmed in single neuron simulations, which lead mostly to either horizontal or vertical orientations, at random positions (Fig 5d). When the network is expanded to 50 neurons, recurrent inhibition forces receptive fields to cover different positions, though excluding border positions, and some neurons have non-cardinal orientations (Fig 5e). With 1000 neurons, receptive fields diversify to many possible combinations of position, orientation and length (Fig 5f).

### High sensitivity to input correlations

Natural images have non-uniform spectral properties, with higher variance at low spatial frequencies [39]. Since Hebbian learning is sensitive to second-order correlations, in order to learn receptive fields driven by higher-order statistics, most studies pre-whiten the input, making the variance uninformative [36]. While there is evidence that the early sensory pathway induces decorrelation across neurons [45], it is unlikely for the input to the visual cortex to be perfectly white.

To analyze the impact of residual second-order correlations, we simulated nonlinear Hebbian learning with natural image patches that have been only approximately whitened. Instead of estimating the whitening filter from input correlation matrix, we used the preprocessing filter from the original sparse coding studies [6, 37], which assumes that natural images possess an ideal power-law energy spectra (see Methods).

**Fig 6:**
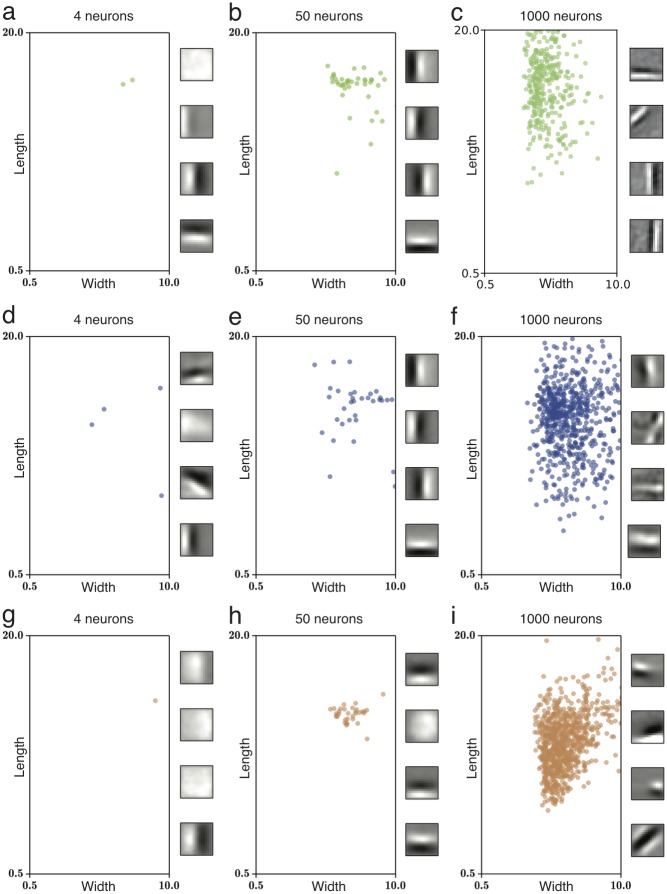
Receptive fields for non-whitened natural images. (**a-i**) Receptive field obtained for network simulations with the quadratic rectifier (top), linear rectifier (center) and *L*_0_ sparse coding (bottom). For few neurons (left and center), the principal components dominate the optimization and receptive fields are nonlocal, since they extend over most of the image patch. For an overcomplete network with 1000 neurons (right), lateral inhibition promotes diversity of receptive fields, including more localized ones. (**insets**) Sample receptive fields developed for each simulation.

In Fig 6, we show the receptive fields learned for non-white inputs through nonlinear Hebbian learning. For networks with few neurons (Fig 6a and 6b), nonlocal receptive fields develop, with shapes similar to the principal components of natural images [6]. It reflects that when second-order correlations are present, these dominate over higher-order statistics, in which case the models we have considered will not reproduce the development of localized oriented filters. However, when considering an overcomplete network with 1000 neurons, smaller receptive fields are learned (Fig 6c). Our optimization framework provides a new perspective on this phenomena. For non-white inputs, second-order correlation dominate the optimization values, making principal components optima. However, when more neurons are added, competition drives the diversification of receptive fields away from the optima, and localized filters with optimization values driven by higher-order statistics can be learned.

We also compare the receptive fields developed for different nonlinearities (Fig 6d–6i). Particularly, the quadratic rectifier appears to develop more elongated filters compared to the linear rectifier network, while the *L*_0_ sparse coding network develops shorter ones. However, these differences across nonlinearities are minor compared to the difference to the receptive fields for white inputs (Fig 4) or compared to the differences observed across different network sizes. Thus, our results suggest that efforts to model receptive field shapes observed experimentally [41, 40, 9, 12] should pay particular attention to network size and input preprocessing, which may have a greater effect than the properties of the particular model.

### Beyond V1 simple cells

Nonlinear Hebbian learning is not limited to explaining simple cells in V1. We investigated if the same learning principles apply to receptive field development in other visual or auditory areas or under different rearing conditions.

**Fig 7:**
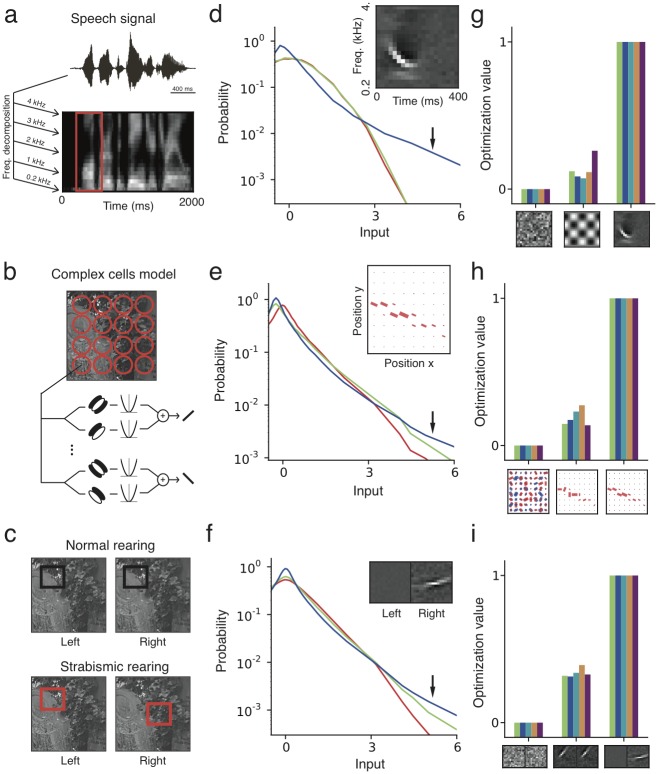
Nonlinear Hebbian learning across sensory modalities. (**a**) The auditory input is modeled as segments over time and frequency (red) of the spectrotemporal representation of speech signals. (**b**) The V2 input is assembled from the output of modeled V1 complex cells at different positions and orientations. Receptive fields are represented by bars with size proportional to the connection strength to the complex cell with the respective position and orientation. (**c**) Strabismic rearing is modeled as binocular stimuli with non-overlapping left and right eye input patches (red). (**d-f**) Statistical distribution (log scale) of the input projected onto three different features for speech (**d**), V2 (**e**) and strabismus (**f**). In all three cases, the learned receptive field (blue, inset) is characterized by a longer tailed distribution (arrows) than the random (red) and comparative (green) features. (**g-i**) Relative optimization value for five nonlinearities (same as in Fig 2), for the three selected patterns (**insets**). The receptive fields learned with the quadratic rectifier nonlinearity (*θ*_1_ = 1., *θ*_2_ = 2.) are the maxima among the three patterns, for all five nonlinearities, for all three datasets.

For auditory neurons [14], we used segments of speech as input (Fig 7a) and observed the development of spectrotemporal receptive fields localized in both frequency and time [2] (Fig 7d). The statistical distribution of input patterns aligned with the learned receptive fields had longer tails than for random or non-local receptive fields, indicating temporal sparsity of responses (Fig 7d). Similar to our simple cell results, the learned receptive fields show higher optimization value for all five effective nonlinearities (Fig 7g).

For a study of receptive field development in the secondary visual cortex (V2) [16], we used natural images and the standard energy model [46] of V1 complex cells to generate input to V2 (Fig 7b). The learned receptive field was selective to a single orientation over neighboring positions, indicating a higher level of translation invariance. When inputs were processed with this receptive field, we found longer tails in the feature distribution than with random features or receptive fields without orientation coherence (Fig 7e), and the learned receptive field had a higher optimization value for all choices of nonlinearity (Fig 7h).

Another important constraint for developmental models are characteristic deviations, such as strabismus, caused by abnormal sensory rearing. Under normal binocular rearing conditions, the fan-in of synaptic input from the left and right eyes overlap in visual space (Fig 7c). In this case, binocular receptive fields with similar features for left and right eyes develop. In the strabismic condition, the left and right eyes are not aligned, modeled as binocular rearing with non-overlapping input from each eye (Fig 7c). In this scenario, a monocular simple cell-like receptive field developed (Fig 7f), as observed in experiments and earlier models [28]. The statistical distributions confirm that for disparate inputs the monocular receptive field is more kurtotic than a binocular one, explaining its formation in diverse models [47] (Fig 7f and 7i).

Our results demonstrate the generality of the theory across multiple cortical areas. Selecting a relevant feature space for an extensive analysis, as we have done with simple cells and natural images, may not be possible in general. Nonetheless, nonlinear Hebbian learning helps to explain why some features (and not others) are learnable in network models [15].

## Discussion

Historically, a variety of models have been proposed to explain the development and distribution of receptive fields. We have shown that nonlinear Hebbian learning is a parsimonious principle which is implicitly or explicitly present in many developmental models [6, 7, 8, 9, 10, 11, 12, 24, 38, 42, 47]. The fact that receptive field development is robust to the specific nonlinearity highlights a functional relation between different models. It also unifies feature learning across sensory modalities: receptive fields form around features with a long-tailed distribution.

### Relation to previous studies

Earlier studies have already placed developmental models side by side, comparing their normative assumptions, algorithmic implementation or receptive fields developed. Though consistent with their findings, our results lead to revised interpretations and predictions.

The similarities between sparse coding and ICA are clear from their normative correspondence [37]. Nevertheless, the additional constraint in ICA, of having at most as many features as inputs, makes it an easier problem to solve, allowing for a range of suitable algorithms [36]. These differ from algorithms derived for sparse coding, in which the inference step is difficult due to overcompleteness. We have shown that regardless of the specific normative assumptions, it is the common implementation of nonlinear Hebbian learning that explains similarities in their learning properties. Since a given normative model may have very different algorithms, as exemplified by the family of ICA algorithms [36], this result is not trivial, and it has previously not been clear how sparse coding and ICA models related to each other at the algorithmic level.

In contrast to the idea that in sparse coding algorithms overcompleteness is required for development of localized oriented edges [37], we have demonstrated that a sparse coding model with a single neuron is mathematically equivalent to nonlinear Hebbian learning and learns localized filters in a setting that is clearly “undercomplete”. Thus differences observed in receptive field shapes between sparse coding and ICA models [40] are likely due to differences in network size and input preprocessing. For instance, the original sparse coding model [37] applied a preprocessing filter that did not completely whiten the input, leading to larger receptive fields (Fig 6).

Studies that derive spiking models from normative theories often interpret the development of oriented receptive fields as a consequence of its normative assumptions [11, 12]. In a recent example, a spiking network has been related to the sparse coding model [12], using neural properties defined ad hoc. Our results suggest that many other choices of neural activations would have given qualitatively similar receptive fields, independent of the sparse coding assumption. While in sparse coding the effective nonlinearity derives from a linear plasticity rule combined with a nonlinear f-I curve, our results indicate that a nonlinear plasticity rule combined with a linear neuron model would give the same outcome.

In order to distinguish between different normative assumptions, or particular neural implementations, the observation of “oriented filters” is not sufficient and additional constraints are needed. Similarly receptive shape diversity, another important experimental constraint, should also be considered with care, since it cannot easily distinguish between models either. Studies that confront the receptive field diversity of a model to experimental data [41, 40, 9, 12] should also take into account input preprocessing choices and how the shape changes with an increasing network size, since we have observed that these aspects may have a larger effect on receptive field shape than the particulars of the learning model.

Empirical studies of alternative datasets, including abnormal visual rearing [47], tactile and auditory stimuli [15], have also observed that different unsupervised learning algorithms lead to comparable receptive fields shapes. Our results offer a plausible theoretical explanation for these findings.

Past investigations on nonlinear Hebbian learning [20, 38] demonstrated that many nonlinearities were capable of solving the cocktail party problem. Since it is a specific toy model, that asks for the unmixing of linearly mixed independent features, it is not clear a priori whether the same conclusions would hold in other settings. We have shown that the results of [20] and [38] generalize in two directions. First, the effective nonlinear Hebbian learning mechanism is also behind other models beyond ICA, such as sparse coding models and plastic spiking networks. Second, the robustness to the choice of nonlinearity is not limited to a toy example, but also holds in multiple real world data. Our approach of identifying generic principles enables us to transfer results from one model, such as orientation selectivity or optimization of higher-order statistics to other models within the general framework. Therefore our insights may contribute to predict the outcome of a variety of developmental models in diverse applications.

### Robustness to normative assumptions

Many theoretical studies start from normative assumptions [7, 9, 11, 37], such as a statistical model of the sensory input or a functional objective, and derive neural and synaptic dynamics from them. Our claim of universality of feature learning indicates that details of normative assumptions may be of lower importance.

For instance, in sparse coding one assumes features with a specific statistical prior [9, 37]. After learning, this prior is expected to match the posterior distribution of the neuron’s firing activity [9, 37]. Nevertheless, we have shown that receptive field learning is largely unaffected by the choice of prior. Thus, one cannot claim that the features were learned because they match the assumed prior distribution, and indeed in general they do not. For a coherent statistical interpretation, one could search for a prior that would match the feature statistics. However, since the outcome of learning is largely unaffected by the choice of prior, such a statistical approach would have limited predictive power. Generally, kurtotic prior assumptions enable feature learning, but the specific priors are not as decisive as one might expect. Because normative approaches have assumptions, such as independence of hidden features, that are not generally satisfied by the data they are applied to, the actual algorithm that is used for optimization becomes more critical than the formal statistical model.

The concept of sparseness of neural activity is used with two distinct meanings. The first one is a single-neuron concept and specifically refers to the long-tailed distribution statistics of neural activity, indicating a “kurtotic” distribution. The second notion of sparseness is an ensemble concept and refers to the very low firing rate of neurons, observed in cortical activity [48], which may arise from lateral competition in overcomplete representations. Overcompleteness of ensembles makes sparse coding different from ICA [37]. We have shown here that competition between multiple neurons is fundamental for receptive field diversity, whereas it is not required for simple cell formation per se. Kurtotic features can be learned even by a single neuron with nonlinear Hebbian learning, and with no restrictions on the sparseness of its firing activity.

Recent studies have also questioned normative explanations for V1 receptive fields by highlighting that these models do not accurately capture the statistics of natural images [49, 50]. The generative models learned for sparse coding or ICA do not generate qualitatively good samples of natural image patches [50]. In particular, the performance in the quantitative criteria that these models are designed to optimize, such as likelihood of the data [50] or higher-order redundancy [49], is sometimes only marginally better than that of simpler models. Further studies are necessary to elucidate more complex models going beyond the two-layer model considered here.

For instance, models of spiking networks learning spatio-temporal patterns have been proposed based on diverse principles such as reward-modulated plasticity [51, 52], novelty-like global factors [53, 54] and temporal correlations [55, 56]. It would be interesting to investigate if generality principles can also shed light on such models. Furthermore, top-down inputs form a substantial part of the incoming signal to sensory areas [57] and it is unclear how they might affect learning and representation in sensory networks. Multi-layered models of probabilistic inference may provide ways to integrate these aspects under a coherent framework for sensory development [58, 59, 60].

Our arguments can be formulated using Marr’s three levels of analysis: the computational level, the algorithmic level and the implementational level [61]. We have argued that the algorithmic level, through nonlinear Hebbian learning, is fundamental in understanding many current models of sensory development, while being consistent with multiple biological implementations and computational goals. Our results show that the models and experimental evidence considered were not sufficient to conclusively discriminate between normative assumptions, indicating indeterminacy at the computational level. Since ultimately one also wants a normative understanding of sensory networks, our results argue for more experimental evidence to be taken into account, requiring more complex models, which in turn shall be described by, or derived from, precise computational objectives, such as probabilistic inference or efficient coding.

### Interaction with input preprocessing and homeostatic mechanisms

The concept of nonlinear Hebbian learning also clarifies the interaction of feature selectivity with input preprocessing. Most studies of receptive field development consider pre-whitened inputs, which may be justified by the evidence that the early sensory pathway decorrelates neural activity [62]. However, we have shown that developmental models are highly sensitive to second-order statistics, and even residual correlations will substantially alter receptive field development. When correlations at low spatial frequencies were present in the input images, nonlinear Hebbian learning models learned nonlocal receptive fields.

In this case, additional mechanisms become necessary to reproduce the development of localized receptive fields as observed in the visual cortex. One possibility is that the competition in overcomplete networks drives the diversify of receptive fields away from principal components, so that neurons become sensitive to higher-order statistics [6]. Another explanation is that the restriction on the arborization of input connections is responsible for local properties of V1 receptive fields [63], in which case localization is not related to higher-order statistics. These considerations demonstrate how alternative input preprocessing can radically change the interpretation of developmental studies, and suggests that more attention should be paid to the preprocessing steps performed in modeling studies. Importantly, it highlights the necessity of more investigations on learning models with robustness to second-order correlations.

In studies of spiking networks, the input is restricted to positive rates, possibly through an on/off representation, as observed in the LGN [63]. In such alternative representations, trivial receptive fields may develop, such as a single non-zero synapse, and additional mechanisms, such as hard bounds on each synaptic strength, *a* ≤ *w*_*j*_ ≤ *b*, may be necessary to restrict the optimization space to desirable features [10].

Instead of constraining the synaptic weights, one may implement a synaptic decay as in Oja’s plasticity rule [35], Δ**w** ∝ **x** *y* − **w** *y*^2^ (see also [64]). Because of its multiplicative effect, the decay term does not alter the receptive field, but only scales its strength. Thus, it is equivalent to rescaling the input in the f-I curve, so as to shift it to the appropriate range (Fig 3). Similar scaling effects arise from f-I changes due to intrinsic plasticity [11, 30, 65] or due to the sliding threshold in BCM-like models, where the effective nonlinearity is modulated by the current weights. Since we have shown that receptive field development is robust to the specific nonlinearity, we expect our results in general to remain valid in the presence of such homeostatic mechanisms. The precise relation between nonlinear Hebbian learning, spiking representations and homeostasis in the cortex is an important topic for further studies.

### Universality supports biological instantiation

The principle of nonlinear Hebbian learning has a direct correspondence to biological neurons and is compatible with a large variety of plasticity mechanisms. It is not uncommon for biological systems to have diverse implementations with comparable functional properties [66]. Different species, or brain areas, could have different neural and plasticity characteristics, and still have similar feature learning properties [67, 68]. The generality of the results discussed in this paper reveals learning simple cell-like receptive fields from natural images to be much easier than previously thought. It implies that a biological interpretation of models is possible even if some aspects of a model appear simplified or even wrong in some biological aspects. Universality also implies that the study of receptive field development is not sufficient to distinguish between different models.

The relation of nonlinear Hebbian learning to projection pursuit endorses the interpretation of cortical plasticity as an optimization process. Under the rate coding assumptions considered here, the crucial property is an effective synaptic change linear in the pre-synaptic rate, and nonlinear in the post-synaptic input. Pairing experiments with random firing and independently varying pre- and post-synaptic rates would be valuable to investigate these properties [27, 69, 70]. Altogether, the robustness to details in both input modality and neural implementation suggests nonlinear Hebbian learning as a fundamental principle underlying the development of sensory representations.

## Methods

### Spiking model

A generalized leaky integrate-and-fire neuron [26] was used as spiking model, which includes power-law spike-triggered adaptation and stochastic firing, with parameters [26] fitted to pyramidal neurons. The f-I curve *g*(*I*) was estimated by injecting step currents and calculating the trial average of the spike count over the first 500 ms. The minimal triplet-STDP model [24] was implemented, in which synaptic changes follow

$$\frac{d}{dt}w\left(t\right)\;=\;A^{+}y\left(t\right){\bar{y}}^{+}\left(t\right){\bar{x}}^{+}\left(t\right)\;-\;A^{-}x\left(t\right){\bar{y}}^{-}\left(t\right)$$ (4)

where *y*(*t*) and *x*(*t*) are the post- and pre-synaptic spike trains, respectively: *y*(*t*) = ∑_*f*_ *δ*(*t* − *t*^*f*^), where *t*^*f*^ are the firing times and *δ* denotes the Dirac *δ*-function; *x*(*t*) is a vector with components $x_{i}\left(t\right)\;=\;\sum_{f}\delta \left(t\;-\;t_{i}^{f}\right)$, where $t_{i}^{f}$ are the firing times of pre-synaptic neuron *i*; *w* is a vector comprising the synaptic weights *w*_*i*_ connecting a pre-synaptic neuron *i* to a post-synaptic cell. *A*^+^ = 6.5 ⋅ 10^−3^ and *A*^−^ = 5.3 ⋅ 10^−3^ are constants, and ${\bar{y}}^{+}$, ${\bar{x}}^{+}$ and ${\bar{y}}^{-}$ are moving averages, implemented by integration (e.g. $\tau \frac{\partial \bar{y}}{\partial t}\;=\;-\bar{y}+y$), with time scales 114.0 ms, 16.8 ms and 33.7 ms, respectively [24]. For estimating the nonlinearity *h*(*y*) of the plasticity, pre- and post-synaptic spike trains were generated as Poisson processes, with the pre-synaptic rate set to 20 Hz.

A linear rectifier *g*(*x*) = *a*(*x* − *b*)_+_ was fitted to the f-I curve of the spiking neuron model by squared error optimization. Similarly, a quadratic function *h*(*x*) = *a*(*x*^2^ − *bx*) was fitted to the nonlinearity of the triplet STDP model. The combination of these two fitted functions was plotted as fit for the effective nonlinearity *f*(*x*) = *h*(*g*(*x*)).

### Sparse coding analysis

A sparse coding model, with *K* neurons *y*_1_, …, *y*_*K*_, has a nonlinear Hebbian learning formulation. The sparse coding model minimizes a least square reconstruction error between the vector of inputs **x** and the reconstruction vector **Wy**, where **W** = [**w**_1_ …**w**_*K*_], and **y** = (*y*_1_, …, *y*_*K*_) is the vector of neuronal activities, with *y*_*j*_ ≥ 0 for 1 ≤ *j* ≤ *K*. The total error *E* combines a sparsity constraint *S* with weight *λ* and the reconstruction error, $E\;=\;\frac{1}{2}||x\;-\;{Wy||}^{2}\;+\;\lambda \sum S\left(y_{k}\right)$. *E* has to be minimal, averaged across all input samples, under the constraint *y*_*j*_ ≥ 0 for all *j*.

The minimization problem is solved by a two-step procedure. In the first step, for each input sample, one minimizes *E* with respect to all hidden units *y*_*j*_

$$\begin{array}{cc} \frac{d}{dy_{j}}E\;=\;0 & \Leftrightarrow \;w_{j}^{T}\left(x\;-\;Wy\right)\;-\;\lambda S^{\prime }\left(y_{j}\right)\;=\;0\\ & \Leftrightarrow \;w_{j}^{T}x\;-\;\sum_{k\neq j}\left(w_{j}^{T}w_{k}\right)y_{k}\;-\;\left|\right|w_{j}{\left|\right|}^{2}y_{j}\;-\;\lambda S^{\prime }\left(y_{j}\right)\;=\;0\\ & \Leftrightarrow \;y_{j}\;+\;\lambda S^{\prime }\left(y_{j}\right)\;=\;w_{j}^{T}x\;-\;\sum_{k\neq j}\left(w_{j}^{T}w_{k}\right)y_{k}\\ & \Leftrightarrow \;y_{j}\;=\;g\left(w_{j}^{T}x\;-\;\sum_{k\neq j}v_{jk}y_{k}\right) \end{array}$$ (5)

where we constrained the vector **w**_*j*_ of synapses projecting onto unit *y*_*j*_ by ||**w**_*j*_||^2^ = 1, defined the activation function *g*(.) = *T*^−1^(.), the inverse of *T*(*y*) = (*y*+*λS*′(*y*)), and defined recurrent synaptic weights $v_{jk}=w_{j}^{T}w_{k}$. For each input sample **x**, this equation shall be iterated until convergence. The equation can be interpreted as a recurrent neural network, where each neuron has an activation function *g*, and the input is given by the sum of the feedforward drive $w_{j}^{T}x$ and a recurrent inhibition term −∑_*k* ≠ *j*_ *v*_*jk*_ *y*_*k*_. To avoid instability, we implement a smooth membrane potential *u*_*j*_, which has the same convergence point [34]

$$\begin{array}{cc} & \tau_{u}\frac{d}{dt}u_{j}\left(t\right)\;=\;-u_{j}\left(t\right)\;+\;\left(w_{j}^{T}x\;-\sum_{k\neq j}v_{jk}y_{k}\left(t\right)\right)\\ & y_{j}\left(t\right)\;=\;g\left(u_{j}\left(t\right)\right) \end{array}$$ (6)

initialized with *u*_*j*_(*t*) = 0.

In the second step, we optimize the weights **w**_*j*_, considering the activations *y*_*j*_ obtained in the previous step. Our derivation follows the approach of the original sparse coding study [6], which is related to the Expectation-Maximization (EM) algorithm, in which at this stage the latent variables (here the activations *y*) are treated as constants, so that $\frac{dy}{dw_{j}}\;=\;0$, and, in particular, $\frac{d}{dw_{j}}S\left(y\right)\;=\;0$. We obtain a standard gradient descent implementation of the least square regression optimization, leading to a learning rule

$$\Delta w_{j}\;\propto \frac{d}{dw_{j}}E\;=\;\left(x\;-\;W^{T}y\right)\;y_{j}\;=\;x\;y_{j}\;-\;w_{j}\;y_{j}^{2}\;-\sum_{k\neq j}w_{k}y_{k}y_{j}$$

The decay term $w_{j}\;y_{j}^{2}$ has no effect, since the norm is constrained to ||**w**_*j*_|| = 1 at each step. For a single unit *y*, the model simplifies to a nonlinear Hebbian formulation, $\Delta w\;\propto \;x\;g(w_{j}^{T}x)$. For multiple units, it can be interpreted as projection pursuit on an effective input, not yet represented by other neurons, $\tilde{x_{j}}\;=\;x\;-\;\sum_{k\neq j}w_{k}y_{k}$, which simplifies to $\Delta w_{j}\;\propto \;{\tilde{x}}_{j}\;g\left(w_{j}^{T}\tilde{x_{j}}\right)$.

There are two non-local terms that need to be implemented by local mechanisms so as to be biologically plausible. First, the recurrent weights depend on the overlap between receptive fields, $w_{j}^{T}w_{k}$, which is non-local. The sparse coding model assumes independent hidden neurons, which implies that after learning neurons should be pair-wise uncorrelated, *cov*(*y*_*j*_, *y*_*k*_) = 0. As an aside we note that the choice $v_{jk}\;=\;w_{j}^{T}w_{k}$ does not automatically guarantee decorrelation. Decorrelation may be enforced through plastic lateral connections, following an anti-Hebbian rule [42, 12], Δ*v*_*jk*_ ∝ (*y*_*j*_−〈*y*_*j*_〉) ⋅ *y*_*k*_, where 〈*y*_*j*_〉 is a moving average (we use *τ* = 1000 input samples). Thus by substituting fixed recurrent connections by anti-Hebbian plasticity, convergence Δ*v*_*jk*_ = 0 implies *cov*(*y*_*j*_, *y*_*k*_) = 0. While this implementation does not guarantee $v_{jk}\;=\;w_{j}^{T}w_{k}$ after convergence, neither does $v_{jk}\;=\;w_{j}^{T}w_{k}$ guarantee decorrelation *cov*(*y*_*j*_, *y*_*k*_) = 0, it does lead to optimal decorrelation, which is the basis of the normative assumption. Additionally we constrain *v*_*jk*_ ≥ 0 to satisfy Dale’s law. Although some weights would converge to negative values otherwise, most neuron pairs have correlated receptive fields, and thus positive recurrent weights.

Second, we ignore the non-local term ∑_*k* ≠ *j*_ **w**_*k*_ *y*_*k*_ *y*_*j*_ in the update rule. Although this approximation is not theoretically justified, we observed in simulations that receptive fields do not qualitatively differ when this term is removed.

The resulting Hebbian formulation can be summarized as

$$\begin{array}{cc} & y_{j}\;=\;g\left(w_{j}^{T}x\;-\;\sum_{k\neq j}v_{jk}y_{k}\right)\\ & \Delta w_{j}\;\propto \;x\;y_{j}\\ & \Delta v_{jk}\;\propto \;\left(y_{j}\;-\;\langle y_{j}\rangle \right)\cdot y_{k} \end{array}$$ (7)

This derivation unifies previous results on the biological implementation of sparse coding: the relation of the sparseness constraint to a specific activation function [34], the derivation of a Hebbian learning rule from quadratic error minimization [35], and the possibility of approximating lateral interaction terms by learned lateral inhibition [42, 12].

### Nonlinearities and optimization value

The optimization value for a given effective nonlinearity *f*, synaptic weights *w*, and input samples *x*, is given by *R* = 〈*F*(**w**^*T*^**x**)〉, where $F\left(u\right)\;=\;\int_{0}^{u}f\left(u^{\prime }\right)du^{\prime }$ and angular brackets indicate the ensemble average over *x*. Relative optimization values in Figs 2b and 5 were normalized to [0, 1], relative to the minimum and maximum values among the considered choice of features *w*, R* = (*R* − *R*_*min*_)/(*R*_*max*_ − *R*_*min*_). The selectivity index of a nonlinearity *f* is defined as *SI* = (〈*F*(*l*)〉 − 〈*F*(*g*)〉)/*σ*_*F*_, where *l* and *g* are Laplacian and Gaussian variables respectively, normalized to unit variance. $\sigma_{F}\;=\;\sqrt{\sigma_{F(l)}\sigma_{F(g)}}$ is a normalization factor, with $\sigma_{F(.)}\;=\;\sqrt{\langle F{\left(.\right)}^{2}\rangle }$. The selectivity of an effective nonlinearity *f* is not altered by multiplicative scaling, $\tilde{f}\left(u\right)\;=\;\alpha f\left(u\right)$, neither by additive constants when the input distribution is symmetric, $\tilde{f}\left(u\right)\;=\;\alpha f\left(u\right)+\beta$. The effective nonlinearities in Fig 2 included the linear rectifier $f\left(u\right)\;=\;\{\begin{array}{cc} 0, & if\;u\;<\;\theta \\ u\;-\;\theta , & if\;u\;\geq \;\theta \end{array}$, the quadratic rectifier $f\left(u\right)\;=\;\{\begin{array}{cc} 0, & if\;u\;<\;\theta \\ (u\text{ − }\theta )(u\text{ − }\theta \text{ − }b), & if\;u\;\geq \;\theta \end{array}$, the *L*_0_ sparse coding nonlinearity $f\left(u\right)\;=\;\{\begin{array}{cc} 0, & if\;u\;<\;\lambda \\ u, & if\;u\;\geq \;\lambda \end{array}$, the Cauchy sparse coding nonlinearity *f* = *T*^ − 1^, where $T\left(y\right)\;=\;\{\begin{array}{cc} 0, & if\;y\;<\;0\\ y\;+\;2\lambda y/(1\;+\;y^{2}), & if\;y\;\geq \;0 \end{array}$, the negative sigmoid *f*(*u*) = 1 − 2/(1 + *e*^ − 2*u*^), a polynomial function *f*(*u*) = *u*^3^, trigonometric functions *sin*(*u*) and *cos*(*u*), a symmetric piece-wise linear function $f\left(u\right)\;=\;\{\begin{array}{cc} 0, & if\;|u|\;<\;\theta \\ |u|\;-\;\theta , & if\;|u|\;\geq \;\theta \end{array}$, as well as, for comparison, a linear function *f*(*u*) = *u*.

### Receptive field learning

Natural image patches (16 by 16 pixel windows) were sampled from a standard dataset [6] (10^6^ patches). Patches were randomly rotated by ±90° degrees to avoid biases in orientation. The dataset was whitened by mean subtraction and a standard linear transformation x* = **Mx**, where **M** = **RD**^ − 1/2^ **R**^*T*^ and 〈**x** **x**^*T*^〉 = **RDR**^*T*^ is the eigenvalue decomposition of the input correlation matrix. In Fig 6, we used images preprocessed as in [6], filtered in the spatial frequency domain by $M\left(f\right)=fe^{-{\left(f/f_{0}\right)}^{4}}$. The exponential factor is a low-pass filter that attenuates high-frequency spatial noise, with *f*_0_ = 200 cycles per image. The linear factor *f* was designed to whiten the images by canceling the approximately 1/*f* power law spatial correlation observed in natural images [39]. But since the exponent of the power law for this particular dataset has an exponent closer to 1.2, the preprocessed images exhibit higher variance at lower spatial frequencies.

Synaptic weights were initialized randomly (normal distribution with zero mean) and, for an effective nonlinearity *f*, evolved through $w_{k+1}\;=\;w_{k}\;+\;\eta \;x_{k}\;f\left(w_{k}^{T}x_{k}\right)$, for each input sample **x**_*k*_, with a small learning rate *η*. We enforced normalized weights at each time step, ||**w**||_2_ = 1, through multiplicative normalization, implicitly assuming rapid homeostatic mechanisms [30, 29]. For multiple neurons, the neural version of the sparse coding model described in Eq 7 was implemented. In Figs 4 and 6, the learned receptive fields were fitted to Gabor filters by least square optimization. Receptive fields with less than 0.6 variance explained were rejected (less than 5% of all receptive fields).

### Receptive field selection

In Fig 2b, the five selected candidate patterns are: random connectivity filter (weights sampled independently from the normal distribution with zero mean), high-frequency Fourier filter (with equal horizontal and vertical spatial periods, *T*_*x*_ = *T*_*y*_ = 8 pixels), difference of Gaussians filter (*σ*_1_ = 3., *σ*_2_ = 4.), low-frequency Fourier filter (*T*_*x*_ = 16, *T*_*y*_ = 32), and centered localized Gabor filter (*σ*_*x*_ = 1.5, *σ*_*y*_ = 2.0, *f* = 0.2, *θ* = *π*/3, *ϕ* = *π*/2). Fourier filters were modeled as *w*_*ab*_ = *sin*(2*πa*/*T*_*x*_) ⋅ *cos*(2*πb*/*T*_*y*_); difference of Gaussians filters as the difference between two centered 2D Gaussians with same amplitude and standard deviations *σ*_1_ and *σ*_2_; and we considered standard Gabor filters, with center (*x*_*c*_, *y*_*c*_), spatial frequency *f*, width *σ*_*x*_, length *σ*_*y*_, phase *ϕ* and angle *θ*. In Figs 4 and 6 we define the Gabor width and length in pixels as 2.5 times the standard deviation of the respective Gaussian envelopes, *σ*_*x*_ and *σ*_*y*_. In Fig 5a, a Gabor filter of size *s* had parameters *σ*_*x*_ = 0.3 ⋅ *s*, *σ*_*y*_ = 0.6 ⋅ *s*, *f* = 1/*s* and *θ* = *π*/3. In Fig 5b and 5c, the Gabor filter parameters were *σ*_*x*_ = 1.2, *σ*_*y*_ = 2.4, *f* = 0.25. All receptive fields were normalized to ||**w**||_2_ = 1. In Figs 4 and 6, the background optimization value was calculated for Gabor filters of different widths, lengths, frequencies, phases *ϕ* = 0 and *ϕ* = *π*/2. For each width and length, the maximum value among frequencies and phases was plotted.

### Additional datasets

For the strabismus model, two independent natural image patches were concatenated, representing non-overlapping left and right eye inputs, forming a dataset with 16 by 32 patches [28]. For the binocular receptive field in the strabismus statistical analysis (Fig 7a), a receptive field was learned with a binocular input with same input from left and right eyes. As V2 input, V1 complex cell responses were obtained from natural images as in standard energy models [46], modeled as the sum of the squared responses of simple cells with alternated phases. These simple cells were modeled as linear neurons with Gabor receptive fields (*σ*_*x*_ = 1.2, *σ*_*y*_ = 2.4, *f* = 0.3), with centers placed on a 8 by 8 grid (3.1 pixels spacing), with 8 different orientations at each position (total of 512 input dimensions). For the non-orientation selective receptive field in the V2 statistical analysis (Fig 7d), the orientations of the input complex cells for the learned receptive field were randomized. As auditory input, spectrotemporal segments were sampled from utterances spoken by a US English male speaker (CMU US BDL ARCTIC database [71]). For the frequency decomposition [14], each audio segment was filtered by gammatone kernels, absolute and log value taken and downsampled to 50 Hz. Each sample was 20 time points long (400 ms segment) and 20 frequency points wide (equally spaced between 0.2 kHz and 4.0 kHz). For the non-local receptive field in the auditory statistical analysis (Fig 7g), a Fourier filter was used (*T*_*t*_ = *T*_*f*_ = 10). For all datasets, the input ensemble was whitened after the preprocessing steps, by the same linear transformation described above for natural images, and all receptive fields were normalized to ||**w**||_2_ = 1.
